# Dega osteotomy for the management of developmental dysplasia of the hip in children aged 2–8 years: results of 58 consecutive osteotomies after 13–25 years of follow-up

**DOI:** 10.1007/s11832-015-0665-9

**Published:** 2015-06-23

**Authors:** Mohamed M. H. El-Sayed, Mohamed Hegazy, Nasef M. Abdelatif, Mohamed A. ElGebeily, Tamer ElSobky, Sean Nader

**Affiliations:** Department of Pediatric Orthopedics, Tanta University, 13 Omar Zafan St, 6th Floor, Tanta, Al Gharbiyah 3111 Egypt; Department of Pediatric Orthopedics, Cairo University, Cairo, Egypt; Department of Pediatric Orthopedics, Bani Suef University, Beni Suef, Egypt; Department of Pediatric Orthopedics, Ain Shams University, Cairo, Egypt; Department of Pediatric Orthopedics, Schön Klinik Vogtareuth, Vogtareuth, Deutschland

**Keywords:** Dega, Pelvic osteotomy, Femoral osteotomy, Developmental dysplasia of the hip, Dysplasia

## Abstract

**Purpose:**

Developmental dysplasia of the hip (DDH) is a term used to cover a broad spectrum of anomalies ranging from mild dysplasia to high-riding dislocations. We report the management of DDH in children using the Dega osteotomy and their long-term follow-up.

**Methods:**

Fifty-eight hips from 48 children younger than 8 years treated using the Dega osteotomy between January 1988 and October 2000 were included in this multcenter study. Both prospective (41 hips) and retrospective (17 hips) cases were included, and follow-up was for a minimum of 13 years. Radiographs were made preoperatively, immediately postoperatively, after 6 weeks or at removal of the spica cast if any, at 6-month intervals and/or as indicated for 3 years postoperatively and then on annual basis until the last follow-up. A single-cut computed tomographic scan was performed for all prospective patients. Special attention was paid to the predictive measures of hip arthrosis and the survival of the hip after Dega osteotomy.

**Results:**

The final clinical outcome was favorable in 44 hips (75.9 %). Eleven hips needed a second surgery (acetabuloplasty and/or arthroplasty) during the follow-up period.

**Conclusions:**

In our pediatric patient population the Dega osteotomy proved to be an adequate measure for the management of this complex condition. The worst complication was avascular necrosis, and all of the affected hips ended with failure (pain, another surgery, or both).

## Introduction

The management of developmental dysplasia of the hip (DDH) in walking children is difficult. In older children, a pelvic osteotomy is needed to achieve a stable concentric reduction. Although much confusion still surrounds the actual procedure of the Dega osteotomy, it is one of the most commonly used osteotomies in the management of DDH. Much of this confusion can be attributed to Dega himself and to his coworkers. In his initial reports on the procedure, Dega [1–3] described two different types of incomplete transiliac osteotomies. However, he never highlighted the important differences between these two procedures, thereby confusing the readers of these reports who were unfamiliar with the exact evolutionary history of his procedure. Dega’s initial osteotomy was first briefly mentioned in a 1964 German publication [2], but it was not until 1969, in a Polish publication [3], that he first referred to this initial osteotomy as a supra-acetabular semicircular osteotomy.

 The objectives of our study were: (1) to evaluate the impact of Dega osteotomy on growth of the acetabulum; (2) to evaluate the remodeling potential of the triradiate after the osteotomy, as monitored by the acetabular index; (3) to evaluate the survival of the treated hips after a minimum of 13 years of follow-up in terms of symptoms and signs of hip arthrosis.

## Patients and methods

Between January 1988 and October 2000, 69 hips (55 patients; 41 unilateral and 14 bilateral) were treated at our medical institutions using the Dega osteotomy. Of these, seven hips were lost during the follow-up period, (5 patients; 3 unilateral and 2 bilateral), and preoperative and early post-operative follow-up X-rays could not be found in another four hips (2 bilateral patients); these 11 hips were excluded from the study. Fifty-eight hips were ultimately included in the study (48 patients; 38 unilateral and 10 bilateral). This was a multicenter study, and the approval of the ethical committees of all participating specialized pediatric orthopedic centers was obtained before data were gathered. Written consent was also obtained from the patients (and/or their parents) for all cases. Of the 58 hips, 48 (82.8 %) were left-sided and ten (17.2 %) were right-sided. The female to male incidence ratio was 2:1.

There were 41 prospective hips (70.7 %; 34 patients) and 17 retrospective hips (29.3 %; 14 patients). Among the included hips were 32 dislocations (Tonnis grades 3 and 4; 55.17 %), 23 subluxated hips (Tonnis grade 2; 39.65 %), and only three dysplastic hips (Tonnis grade 1; 5.18 %) (Table 1). Previous management for the studied hips included failed trial of closed reduction (7 hips; 12 %), open reduction through the medial approach (9 hips; 15.5 %), failed open reduction and capsulorrhaphy through an anterior approach (10 hips; 17.2 %), and a past history of Salter pelvic osteotomy (2 hips; 3.4 %). Of the included hips, 38 were virgin-neglected hips (65.5 %) with no past history of prior treatment; for the remaining 20 hips (34.5 %), at least one of the management options described above had been previously attempted. The ‘bikini’ skin incision (modified Smith–Peterson) was used in all prospective cases. The Dega osteotomy was performed as described by Dega in his original paper [1–3]; this was ensured under strict fluoroscopic guidance. A tricortical iliac crest graft or a fashioned graft from the femoral shortening-osteotomy was then inserted at the osteotomy site. Femoral shortening was performed in 51 hips (87.9 %). Stabilization of the femoral osteotomy was performed using small dynamic compression plates in 45 hips (88.2 %) and an angled blade plate in six hips (11.8 %). A hip-spica cast was applied immediately after surgery for all the hips with a femoral shortening; the hip was immobilized for at least 4–6 weeks and the hip spica removed only after healing of the osteotomy site. When no femoral shortening was performed, no casting was needed. In bilateral cases, surgery on the second hip occurred 6 weeks after the first surgery.

**Table 1:** Preoperative findings of hip dysplasia according to Tonnis classification

Tonnis grades	Number (%) of hips affected
Grade 1	3 (5.18)
Grade 2	23 (39.65)
Grade 3	12 (20.69)
Grade 4	20 (34.48)
Total	58 (100)

**Fig. 1:**
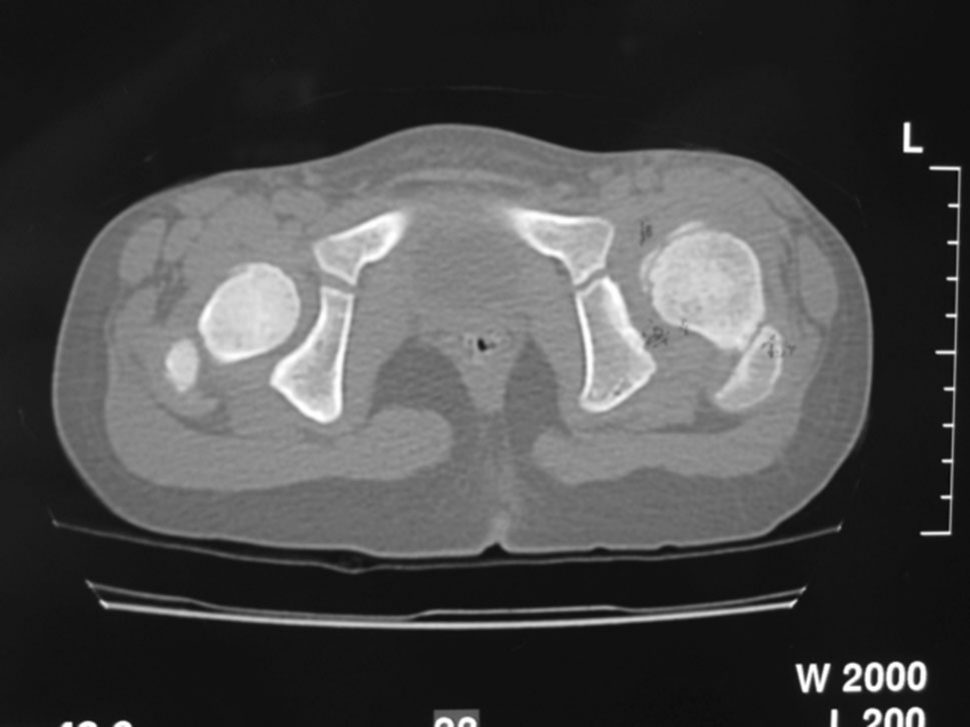
A single-cut computed tomography scan is performed postoperatively to check the adequacy of reduction

Radiographs [traditional anterior-posterior (AP) and lateral views] were made preoperatively, immediately postoperatively, after 6 weeks or at removal of the spica cast if any, at 6-month intervals (ordinary cases) and/or as indicated (complicated cases) for 3 years postoperatively, and then on an annual basis until the last follow-up. A single-cut computed tomographic scan (CT) was performed for all prospective patients to check the adequacy of the reduction within 1 week after the surgery (Fig. 1). Dislocations of the hip were graded using the Tonnis classification system [4]. Other radiographic parameters used in this study included the caput-collum-diaphyseal (CCD) angle [4], the acetabular index (AI) [5], the acetabular angle of Sharp [6], the ACM (points on the pelvic radiograph) angle of Idelberger and Frank (to evaluate the depth of the acetabulum) [7], the center-edge angle of Wiberg (CEA) [8], the migration percentage described by Reimers (MI) [9], disruption of the Shenton line, and the grade of avascular necrosis (AVN) of the femoral head (evaluated using the Salter criteria [10]), In addition, the severity of degenerative arthrosis was assessed according to the system of Tönnis [4] into: Grade 0, indicating no evidence of arthrosis; Grade 1, small spurs with no joint space narrowing; Grade 2, osteophytes with some joint space narrowing; Grade 3, complete loss of the joint space. The final radiographic outcome was evaluated according to the Severin classification [11]. Instead of calculating the summarized hip factor, the CCD angle, AI, acetabular angle, CEA, and summarized hip factor were graded according to the age-adjusted classification system of the German Association of Orthopedics and Trauma Surgery [4, 12, 13], which enabled us to compare the measurements for the different age groups. In the classification system, Grade 1 indicates normal; Grade 2, slightly abnormal; grade 3, severely abnormal; Grade 4, extremely abnormal.

The functional and radiographic results were evaluated at pre-determined time-points and at the final follow-up visit. All patients underwent a final clinical examination and completed a questionnaire which included the Merle d’Aubigné and Postel score [14] as well as the Harris hip score [15]. Failure was defined as a true revision or a Merle d’Aubigné score of <13 points and/or a Harris hip score of <70 points. Barrett’ s modification of McKay’s criteria was also used for the final clinical assessment of the postoperative results [16].

The *p* value for the non-crossing survival curves was calculated using the log-rank test, and the *p* value for the crossing curves was calculated using the Wilcoxon test. Correlation and significance between variables were calculated using the Chi square test, Fisher test, or *t* test.

## Results

The average operating time was 120 (range 90–145) min. The average total intraoperative and postoperative blood loss was 200 (range 125–390) ml. The Dega osteotomy alone was performed in seven hips (12 %), the Dega osteotomy with femoral shortening (FS) was performed in two hips (3.5 %), and the Dega osteotomy with open reduction, capsulorrhaphy, and FS were performed in 49 hips (84.5 %) (Table 2). The age of the patients at the time of surgery ranged from 25 to 90 (mean 48.8) months. The follow-up period ranged from 158 to 302 (mean 199) months. Before the index surgery and based on the Salter criteria of AVN, four of the studied hips showed AVN changes as a sequel of the previous treatments (1 hip after failed closed reduction and 3 hips after open reduction).

**Table 2:** The different surgical procedures performed in the patient population included in this study

Type of procedure performed	Number (%) of hips
Dega alone	7 (12)
Dega + FS	2 (3.5)
Dega + OR + CAP + FS	49 (84.5)
Total number of hips	58 (100)

*FS* Femoral shortening,* OR* open reduction,* CAP* capsulorrhaphy

**Fig. 2:**
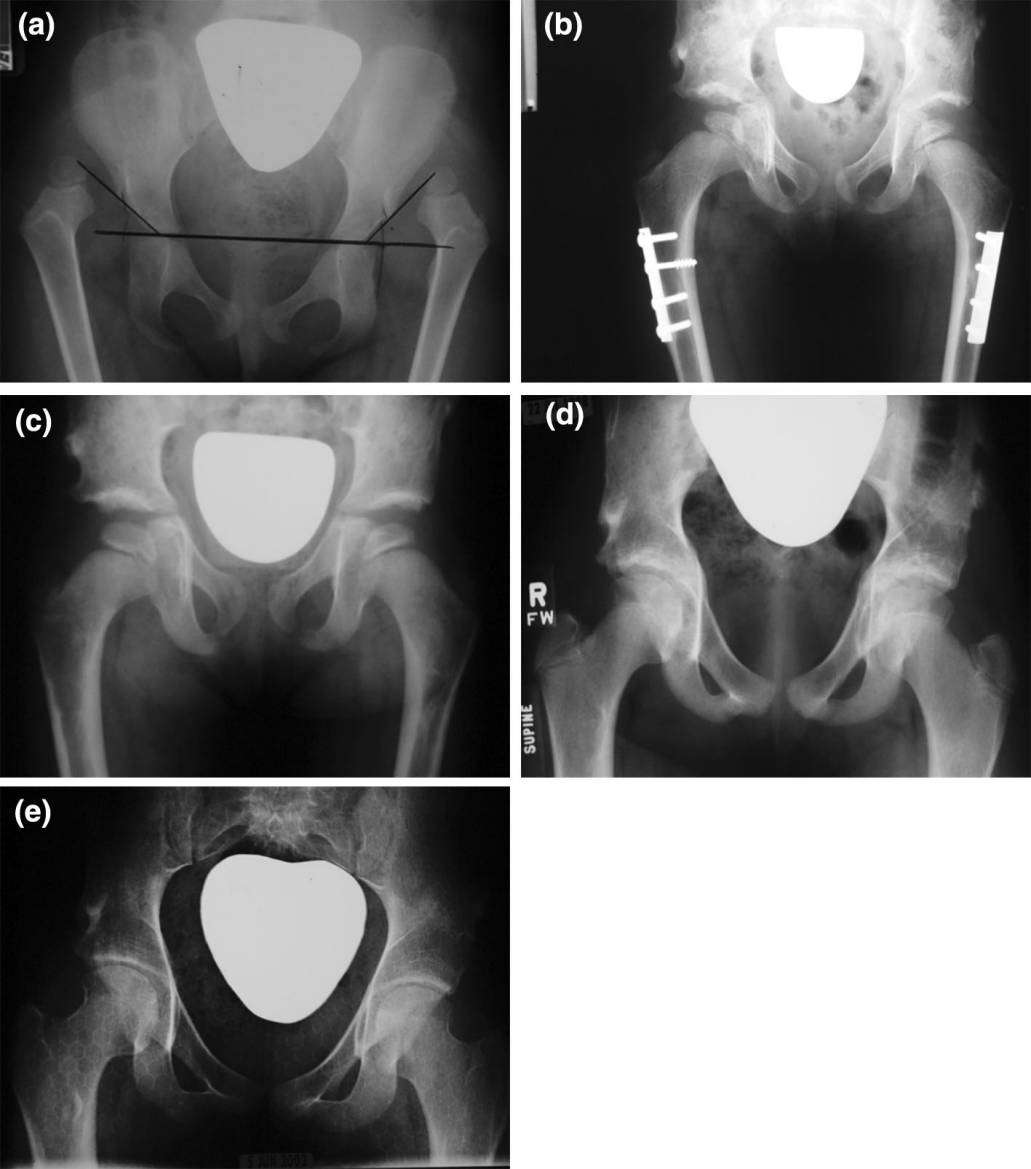
**a** X-rays of young girl with bilateral dysplasia of the hip (DDH) (Tonnis Grade 4) who underwent surgery at 3 years and 6 months of age.** a** Preoperative X-ray anterior-posterior (AP) view, **b** post-operative AP radiograph at 2 years of follow-up after bilateral open reduction (OR), capsulloraphy (Cap), Dega osteotomy, and femoral shortening (FS), **c** AP radiograph at 6 years of follow-up after plate removal, with excellent head coverage and full range of motion, **d** post-operative AP radiograph at 12 years of follow-up, **e** postoperative AP view at 21 years of follow-up with both radiographic and clinical excellent results and no signs of hip arthrosis

Barrett’s modification of McKay’s criteria, which is commonly accepted as the most useful evaluation scheme, was used for the final clinical evaluation of the studied hips. Based on these criteria, 19 hips (32.8 %) were stable and painless, with a full range of motion and negative Trendelenberg sign; these were considered to be excellent results (Fig. 2a–e). There were 25 hips (43.1 %) with a negative Trendelenberg sign but which showed a mild restriction of movement (slight loss of the normal hip range of motion); these were graded clinically as a good result. Four hips (6.9 %) were found to have a moderate stiffness and a positive Trendelenberg sign, although they were stable and painless; these were graded as a fair result. Finally, ten 10 hips (17.2 %) were graded as a poor result because they were ultimately unstable, painful, and/or needed further surgical interference (Table 3). There was a statistically significant correlation between the final clinical outcome and the age of the patients at the time of surgery, with the younger the age of the patient at the time of the index surgery, the better the final clinical outcome. The correlations between the final clinical outcome and the Tonnis preoperative grade of hip dislocation, as well as the final radiographic outcome were also found to be significant.

**Table 3:** The final clinical outcome based on Barret’s modification of McKay’s criteria [16]

Final clinical outcome	Number (%) of patients
Favorable (44 hips)	
Excellent	19 (32.8)
Good	25 (43.1)
Unfavorable (14 hips)	
Fair	4 (6.9)
Poor	10 (17.2)
Total	58 (100)

The questionnaire, which included the Merle d’Aubigné and Postel score [14] and the Harris hip score [15] revealed failure of the surgery for 15 hips (25.8 %).

Att the final follow-up examination and according to the age-adjusted classification system of the German Association of Orthopedics and Trauma Surgery, there were 22 Grade 1 hips (37.9 %), indicating normal movement; 22 Grade 2 hips (37.9 %), considered to indicate slightly abnormal condition; five Grade 3 hips (8.7 %), indicating severely abnormal hips; nine Grade 4 hips (15.5 %), indicating an extremely abnormal condition.

The CEA of Wiberg could not be measured in the majority of cases preoperatively because there were 32 dislocated hips. Immediate postoperative values ranged from 9° to 33°, with a mean of 26°. At the final follow-up examination, the values of the CEA showed further improvement and ranged from 15° to 46°, with a mean of 34° (Table 4). Other important radiographic measurements are also shown in Table 4, while the final radiographic evaluation according to the Severin classification [11] is shown in (Table 5).

**Table 4:** Radiographic evaluation of the studied cases

Radiographic parameter	Preoperative		Immediate postoperative		Final follow-up		Significance
	Range	Mean	Range	Mean	Range	Mean	
Acetabular index (AI)	32–60	39	9–29	18	20–36	25	*
Acetabular angle of Sharp (°) [6]	30–61	52	28–50	39	30–51	40	*
Center-edge angle (CEA) of Wiberg (°) [8]	–	–	9–33	26	15–46	34	–
Remier’s extrusion index	–	–	−0.33±0.25	−0.21	−0.22±0.48	+0.19	–

* *p* value ≤ 0.05 was considered significant

**Table 5:** The final radiographic outcome according to the Severin classification system [11]

Severin classification	Number (%) of hips
Ia	20 (34.6)
Ib	19 (32.7)
II	3 (5.2)
III	1 (1.7)
IVa	2 (3.4)
IVb	11 (19)
V	2 (3.4)
VI	–

## Complications

**Fig. 3:**
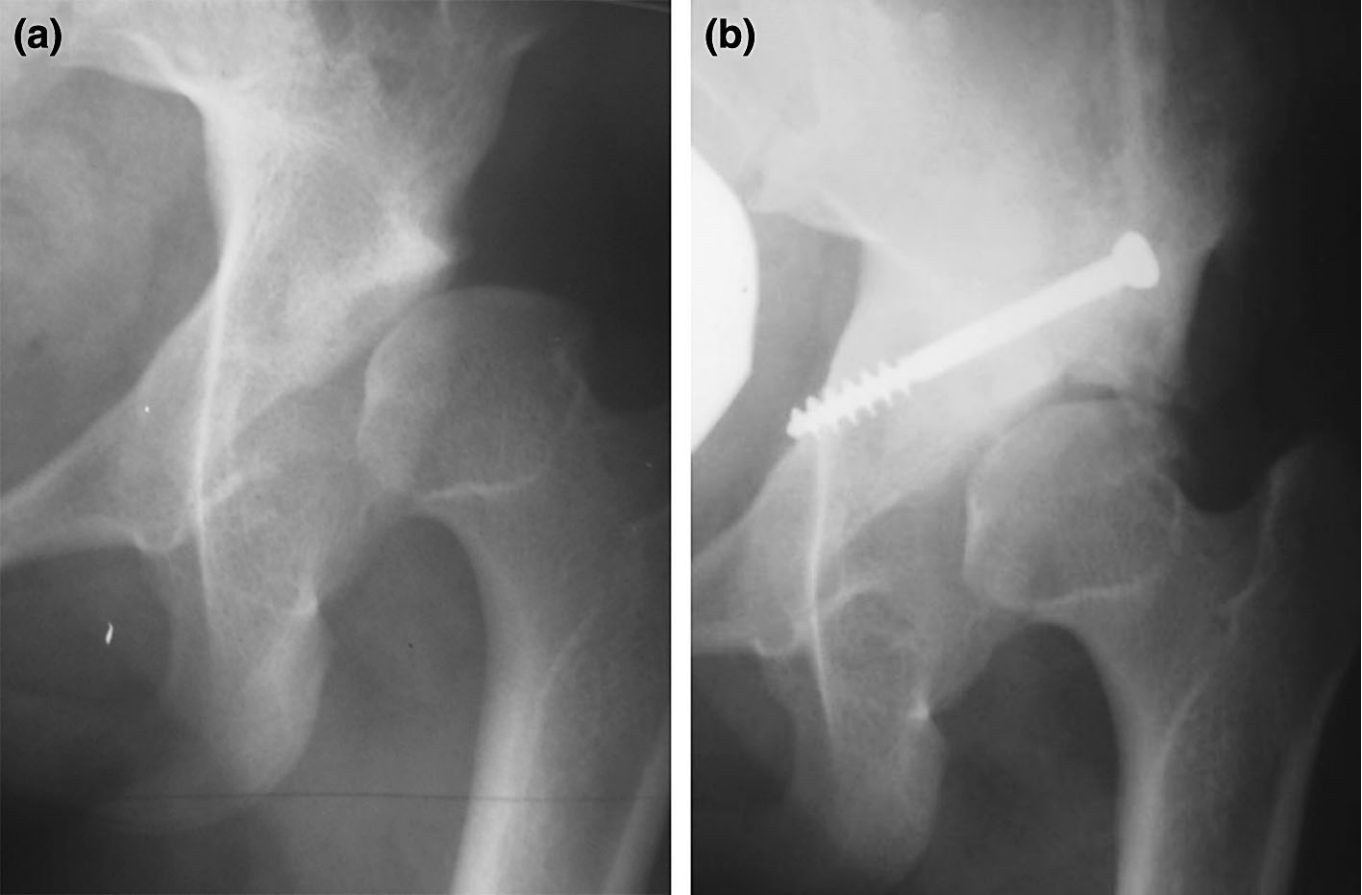
**a** Young female patient with left-sided DDH after open reduction, Cap, Dega, and FS at the age of 4 years and 8 month. She suffered from pain that progressively increased. At 11 years postoperatively, the pain was intolerable and incapacitated the child, and the femoral head was subluxated. **b** A Chiari medial displacement osteotomy was performed to increase the head coverage after 141 months of follow-up. The pain improved but the range of motion was still limited. The hip was considered a failure, and the patient is still under observation

Eight hips (13.7 %) suffered from AVN at the final follow-up examination, including the four cases with preoperative AVN. It was noted that the four new hips with AVN after the index surgery were not virgin cases, and all had a past history of previous open reduction. Residual acetabular dysplasia was found in five hips (8.6 %). Eleven hips (18.9 %) required another surgery during the follow-up period: Dega osteotomy was redone in one case at 25 months postoperatively; Chiari medial displacement osteotomy was performed in two hips at 141 and 155 months, respectively (Fig. 3a, b), a modification of Steel triple innominate osteotomy was performed in four hips at 180, 195, 201, and 211 months of follow-up, respectively, and four total hip arthroplasties took place at 204, 231, 245, and 293 months of follow-up, respectively. Epiphysiodesis of the greater trochanter (2 hips) and distal and lateral transfer of the greater trochanter (4 hips) were also performed in addition to the secondary pelvic osteotomy. All of these hips were considered to be failures. Pain occurred in 14 hips (24.1 %) and led to surgery in 11 hips; in three hips the ain was tolerated by the patient, did not interfere with daily life activity, was managed adequately by conservative measures, and needed no further surgical intervention. The complications encountered in this study are shown in Table 6). It was noted that more than one complication took place in the same hip.

**Table 6:** The complications encountered in this study

Complication	Number (%) of patients
Injury of superficial cutaneous nerve of the thigh	4 (6.8)
Superficial wound infection	2 (3.4)
Fracture of the shaft of the femur after spica removal	2 (3.4)
Limb length discrepancy of <3 cm	8 (13.8)
Avascular necrosis	8 (13.8)
Pain	14 (24.1)
Residual acetabular dysplasia	5 (8.6)
Need for another surgery	11 (18.9)
Positive trendelenberg test	5 (8.6)

At the final follow-up period, 44 hips (75.8 %) had a survival with favorable outcome (excellent or good results). In terms of the degree of hip joint arthrosis at the final follow-up examination, 29 hips (50 %), were considered normal and showed no signs of hip arthrosis (Grade 0); in ten hips (17.2 %) there were small spurs, with no joint space narrowing and slight abnormality (Grade 1); in 14 hips (24.2 %), joint space narrowing was found in addition to osteophytes (Grade 2); in only five hips (8.6 %) was severe hip arthrosis found, with almost total loss of the joint space (Grade 3 arthrosis) (Table 7). Based on radiographic evidence, 39 of the studied hips (67.2 %) survived with either completely normal radiographs or slight abnormalities, without joint space narrowing.

**Table 7:** Presence of hip arthrosis at the final follow-up examination

Hip arthrosis	Number (%) of hips
Grade 0	29 (50)
Grade 1	10 (17.2)
Grade 2	14 (24.2)
Grade 3	5 (8.6)
Total number	58 (100)

Note that; for the hips that needed another surgery, the final follow-up examination was the preoperative findings before the second surgery. In addition, all those hips were considered as failure

## Discussion

In DDH the harmonious association between the growth of the acetabulum, represented by the growth of the triradiate cartilage, and the ossific nucleus of the femoral head is shattered. [16–18] Although the Dega technique is considered to be one of the most common osteotomies performed by pediatric orthopedic surgeons, our review of the literature revealed a paucity of English-language publications on its use in patients with DDH [17–19]. In addition, long-term or even middle-term follow-up studies are lacking. We report here the clinical and radiographic end results of the management of Dega osteotomy in 58 DDH hips, after a mean follow-up of 199 months. The long follow-up period is of special importance since it has been estimated that degenerative joint disease of the hip secondary to dysplasia, subluxation, and/or dislocation occurs in 20–50 % of affected hips [20]. In our pediatric patients, the growth of the acetabulum took place after the Dega osteotomy was performed and was checked using the AI; this control revealed a significant improvement from a mean preoperative AI of 39° to an immediate postoperative mean AI of 18°. This significant improvement in the coverage of the proximal femoral epiphysis was maintained by the remodeling potential of the acetabulum in the form of the triradiate cartilage in this age group. These findings are comparable to the results of Reichel and Hein [21], who recorded an improvement in 18 of their 40 patients, and our measurements are also comparable to those of Al-Ghamdi et al. [17], El-Sayed et al. [18], Grudziak and Ward [22], and Karlen et al. [23]. Although the latter authors used the Dega osteotomy for management of neuromuscular disorders, the magnitude of correction of the AI in their patients were comparable to that of the DDH group of patients. It has been proposed that ‘normal’ values for the AI range from 33° to 40°, while angles of >47° are characteristic of patients with hip dysplasia [17]. We used the AI to monitor our patients as it has been found to be the most useful tool to denote the growth potential of the acetabulum; in our study, it improved to near normal values in patients with favorable outcomes. This result supports the notion of acetabular growth after stable concentric reduction and verifies the growth potential in this particular age group.

The CEA in this study improved to a mean immediate postoperative value of 26°; this value further improved during the follow-up period, achieving a mean of 34° . These results are comparable to those of similar studies reported in the literature [17–20]. This angle is considered to be of particular importance since it is believed to be correlated to the risk of developing hip arthrosis. In our study, hips with favorable CEA (Ia, Ib, II) were found to have very good survival at the end of the follow-up period, with no evidence of arthrosis. These results are also comparable to those reported in the literature [17, 24]. Furthermore, hips with unfavorable CEA (III–V) showed a less favorable outcome and variable degrees of hip arthrosis (Grades 2 and 3).

AVN is the most dreaded complication in DDH management programs, and the rate of AVN affection among our pediatric patient cohort is comparable to published results [16–20]. Moreover, all of the hips suffering from AVN ultimately required a second surgery (acetabuloplasty and/or arthroplasty) before the end of the follow-up period. We believe that the differences in the rate of AVN reported in various studies can be attributed to the hips included in the study: some studies included only virgin hips, others included only patients with a previous history of attempts of closed reduction, and some series included all patients with DDH, even those with a history of failed open reduction (as in our study). Based on our final results, we believe that the rate of AVN in our series was affected by capsulorrhaphy, as suggested by other authors, but we support the claim that this might correlate directly to the type of pelvic osteotomy, as suggested by other authors, due to premature closure of the triradiate which might also lead to residual acetabular dysplasia. [23–25].

Finally, there might be still controversy about the upper age limit for reduction in neglected cases of DDH. We believe that as long as the triradiate cartilage is still open and growing, with the possibility of achieving a stable concentric reduction, there is always be a chance of favorable outcome when the patient is in good expert hands. There were no complications among the patients in our study in terms of the flexibility of the triradiate cartilage, and the magnitude of correction reached up to 38° in the studied age group.

To summarize, the Dega osteotomy is a versatile osteotomy procedure which can be tailored precisely to an individual patient’s need. We found it to be a safe, simple, and adequate procedure in our series of 58 hips for the management of neglected DDH patients with comparable complication rates. We also suggest that the combination of an open reduction, capsulorrhaphy, femoral shortening, and Dega osteotomy is an adequate single-stage surgery in neglected DDH patients of this age group even after a failed trail of open reduction or a previous pelvic osteotomy. Femoral shortening in this age group is of critical importance; among our patients, it facilitated easy reduction and reduced the tension on the proximal femoral epiphysis. We observed that good early postoperative results had the tendency to persist and that the first surgery should be considered as the golden chance to improve the affected hips. We believe that good long-term results can be achieved if the acetabulum can be restored to an up-to-normal configuration, without AVN incidence. In addition, poor preoperative findings (AVN in particular) have the tendency to persist or even worsen during the course of the postoperative follow-up. However, a few patients with good initial results may still develop residual acetabular dysplasia later in life which would then require further surgical intervention.

The limitations of this study include (1) the respective nature of some cases; (2) the need for a longer follow-up period up to skeletal maturity; (3) the need for a multicenter comparable study with various types of pelvic osteotomies. Despite these limitations, we feel that this study adds to the general knowledge of the field and provides valuable interesting information on one of the most commonly used osteotomies for the management of neglected DDH patients younger than 8 years of age, as the results are encouraging.
